# Characteristics of COVID-19 Infection in a Hospitalized Autoimmune Hepatitis Patient

**DOI:** 10.3390/pathogens11091054

**Published:** 2022-09-16

**Authors:** Vanessa Duarte da Costa, Wilian Jean Wiggers, Claudia Alexandra Pontes Ivantes, Rodrigo Jardim Monteiro da Fonseca, Alberto Martín Rivera Dávila, Otacilio C. Moreira, Beatriz Iandra da Silva Ferreira, Vanessa Salete de Paula, Lucas Lima da Silva, Alanna Calheiros Santos, Livia Melo Villar

**Affiliations:** 1 Brazilian Reference Laboratory of Viral Hepatitis, Oswaldo Cruz Institute, Avenida Brasil, 4365, Manguinhos, Rio de Janeiro 21040-360, Brazil; 2 Service of Gastroenterology, Hepatology and Liver Transplantation, Hospital Nossa Senhora das Graças, R. Alcides Munhoz, 433, Mercês, Curitiba 80810-040, Brazil; 3 Computational and Systems Biology Laboratory, Graduate Program in Biodiversity and Health, Oswaldo Cruz Institute, Avenida Brasil, 4365, Manguinhos, Rio de Janeiro 21040-360, Brazil; 4 Molecular Virology Laboratory, Oswaldo Cruz Institute, Avenida Brasil, 4365, Manguinhos, Rio de Janeiro 21040-360, Brazil

**Keywords:** SARS-CoV-2, COVID-19, autoimmune hepatitis

## Abstract

The coronavirus disease 2019 (COVID-19) pandemic, caused by severe acute respiratory syndrome coronavirus-2 (SARS-CoV-2), has become a major public health worldwide. Hepatic dysfunction has been seen in patients with COVID-19 and could be related to a viral cytopathic effect, an exacerbated immune reaction, or drug-induced liver damage. Currently, routine modification of immunosuppressive therapy in patients with autoimmune hepatitis (AIH) before and after SARS-CoV-2 infection remains an important topic to be discussed. However, there is little evidence about this thematic to support any recommendation. Here, we described a case report in which the use of an immunosuppressive drug by a patient with diagnosed AIH might have influenced the COVID-19 clinical course with altered laboratory hematological and biochemical parameters during infection.

## 1. Introduction

The administration of immunosuppressive drugs could alter clinical and laboratory characteristics for patients with AIH and possibly result in a poorer outcome with the evolution of COVID-19 to a more severe disease. Previous studies have described the risk of acquiring SARS-CoV-2 infection for patients with autoimmune diseases, especially those being treated with immunomodulators [1,2]. COVID-19 pandemic has reached approximately 551 million individuals worldwide [3]. The main human organ compromised by SARS-CoV-2 infection is the lung, but given the expression of angiotensin-converting enzyme 2 (ACE2) receptor in cholangiocytes, the liver may also be a potential target [4,5]. Xie et al. (2020) [6] indicated that liver injury is common in COVID-19 patients not admitted to the intensive care unit and that the lung computed tomography (CT) score of the liver-injury group was significantly higher than the group with non-liver injury. Patients with chronic liver disease (CLD) have several mechanisms of immune dysfunction that can result in an increased inflammatory response during viral infection. This immune impairment correlates with reduced macrophage activation, lymphocyte frequency and proliferation, and neutrophil function [7]. In addition, in COVID-19 patients, the following autoimmunological diseases were also observed: Guillain-Barre Syndrome, autoimmune hemolytic anemia, and systemic lupus erythematosus.

We report here a case of a patient with pre-existing AIH in treatment with an immunosuppressive antimetabolite who had positive results for molecular tests during the COVID-19 pandemic. Pre-existing CLD with immune system suppression might have been related to altered laboratory hematological and biochemical parameters during SARS-CoV-2 infection.

## 2. Results

### Case Report

**Figure 1:**
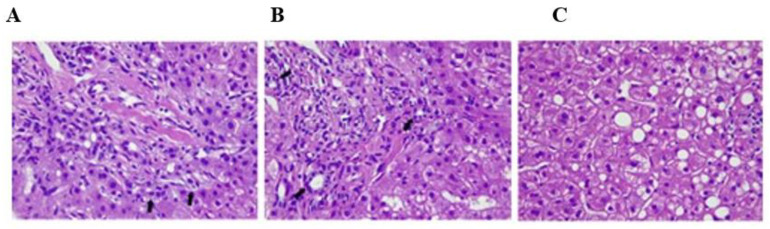
Liver tissue histological sections. (**A**) Inflammatory infiltrate composed of lymphocytes, occasional plasma cells, and eosinophils. The infiltrate extends beyond the limiting lamina (arrow) and is associated with necrose foci, (**B**) portal spaces demonstrate mild bile duct proliferation (arrow), and (**C**) lobule histological findings are characterized by mild macrogoticular steatosis.

The patient was a 33-year-old female who had a definitive diagnosis of AIH in 2019. Before the COVID-19 pandemic, a liver biopsy revealed fibrosis grade 2 and a portal inflammatory infiltrate with ductular reaction and biliary interface (Figure 1). In 2020, she started a follow-up with a hepatologist at Nossa Senhora das Graças Hospital in Paraná/Brazil. Magnetic resonance imaging (MRI) and abdominal ultrasound demonstrated mild hepatic steatosis. The patient reported a contact with a COVID-19-infected family member and a clinical condition of dyspnea in December 2020 that suggested COVID-19. Anamnesis revealed the use of an immunosuppressive drug named azathioprine. She had a history of obesity (BMI: 47.71 kg/m^2^), jaundice, and glycemic alteration. The patient reported being a non-smoker. Referring to vaccine administration, she stated that she had already been vaccinated against hepatitis A and B and tuberculosis. A vaccine against COVID-19 was not available in Brazil at that time.

Molecular diagnosis indicated that she had detectable SARS-CoV-2 RNA with N1, N2, and E gene-cycle threshold (Ct) values of 27.9, 31.4, and 31.3, respectively. Referring to viral load quantification, RT-qPCR for the SARS-CoV-2 N2 target indicated a viral load of 1014 IU/mL (3.01 Log IU/mL). SARS-CoV-2 genotyping by high-throughput nanopore sequencing demonstrated that patient was infected by lineage B.1.1.28.

Her laboratory tests demonstrated normal levels of hemoglobin (14.2 g/dL), total bilirubin (0.5 mg/dL), aspartate aminotransferase (36 U/L), glucose (94 mg/dL), serum urea (21 mg/dL), and creatinine (0.7 mg/dL). Referring to serological tests, HBsAg, anti-HBc, anti-HBs, and anti-HCV were non-reactive. Biochemical and hematological altered parameters in comparison to reference values indicated conditions of leukopenia (3120 leukocytes/mm^3^), lymphopenia (936 lymphocytes/µL), and thrombocytopenia (142.000 platelets/mm^3^). Elevated levels of alanine aminotransferase (49 IU/L) and C-reactive protein (12.8 mg/dL) were also observed (Table 1). In addition, a lung CT demonstrated interstitial infiltrate.

## 3. Discussion

To date, little is known about the clinical and laboratory characteristics of patients with AIH and COVID-19. The challenge remains in obtaining liver tissue and detecting viral infection throughout the short period of active respiratory illness. Elevated rates of hospitalization but with the same rates of mortality for patients with AIH were evidenced compared to non-CLD [1,8]. We reported a COVID-19 moderate case from a patient previously diagnosed with AIH and submitted to 13 days of hospitalization and continuous use of a nasal oxygen catheter. Cases of AIH triggered by the COVID-19 (Oxford/Covishield) vaccination had already been reported [9]. A 38-year-old female and a 62-year-old male had occurrences of jaundice following Covishield vaccine administration. Since the patient of the present case had already been jaundice-justified by AIH and was not vaccinated since no vaccine was available in Brazil at the time of infection, our data could not support the evidence of potential side effects after vaccination. Most patients with AIH are submitted to a permanent immunosuppressive therapy, which may represent a higher risk of acquiring bacterial and/or viral infections [10]. In general, patients with autoimmune liver diseases are characterized by an increased risk of infections given the iatrogenic effect of immunosuppressive drugs which may complicate the SARS-CoV-2 infection outcome [11,12]. Other reports suggest that immunosuppression attributed to drugs may lead to an increased susceptibility to SARS-CoV-2 infection and viral-clearance extension [13,14]. In the clinical case published by Sagnelli et al. (2020) [15], an 82-year-old Caucasian woman had negative prognostic factors (older age, chronic disease, and arterial hypertension), but also maintained the use of tocilizumab and corticosteroid which were associated with the control of severe pneumonia caused by SARS-CoV-2, supporting the role of tocilizumab in controlling severe COVID-19. In the present report, the administration of an immunosuppressor might have suggested a poor evolution of SARS-CoV-2 infection; however no studies had described a possible azathioprine performance in control of SARS-CoV-2 infection. For AIH, the administration of immunosuppressive drugs requires a close monitoring of SARS-CoV-2 infection since their use enhances the risk of severe COVID-19 [16,17]. Presently, EASL, AASLD, and APASL guidelines suggest vast caution in withdrawing immunosuppression in AIH patients with COVID-19, as this could lead to hepatitis outbreaks [18]. Given that assumption, viral RNA was detected when patient was hospitalized with symptoms of dyspnea. In November 2021, the patient still reported severe symptoms corresponding to shortness of breath with a necessity forphysiotherapy. In addition, it was observed that fibrosis evolved to grade 3 (moderate to advanced) in October 2021 while performing at grade 2 in 2019. Recent research from Liu et al. (2022) [19] highlighted that assessing liver fibrosis scores could be an important tool in identifying high risk of developing severe COVID-19 for patients with coexisting COVID-19 and CLD. Regarding the SARS-CoV-2 B.1.1.28 lineage genotyped by high-throughput nanopore sequencing, a Brazilian study from Silva et al. (2021) [20] indicated that this lineage had emerged in February 2020 in São Paulo, Brazil and rapidly spread to other Brazilian states. No association between this lineage and severe COVID-19 was described for CLD patients.

Considering hematological results, several studies have reported decreases in lymphocyte counts in peripheral blood of COVID-19 patients [21,22,23] as observed in the present study. Hematological findings related to lymphopenia had already been observed for 84.6% (11/13) of COVID-19 severe patients which can be mainly associated to significantly decreased T-cells absolute counts, especially CD8+ T cells [22]. When involving hepatic injury in patients with COVID-19, other study from Li et al. (2020) [24] suggested that the occurrence of lymphopenia was independently associated with liver injury and that a cytokine storm might be the main mechanism for inflammatory response. Another study showed that it was unclear whether laboratory test alterations such as disseminated intravascular coagulation (thrombocytopenia) are associated with liver injury caused by the virus or only represent an independent risk factor for previous hepatitis patients [25].

As expected, increased levels of alanine aminotransferase and C-reactive protein were found in this study. Elevated serum levels of alanine aminotransferase were identified in almost 20% of patients with non-severe COVID-19 disease [26,27]. Liver biochemistry abnormalities in COVID-19 are characterized by mild (1–2 times the upper limit of normal) elevations of serum alanine aminotransferase in 29–39% of patients [28,29]. In the present case, this biochemical marker was also slightly elevated for the hospitalized autoimmune hepatitis patient with moderate COVID-19. Hepatitis with elevation of serum alanine transaminases has been reported to occur in up to 50% of SARS-CoV-2-infected patients [25,30]. Increased levels of C-reactive protein in the liver-injury group compared to the group without hepatic damage (31.1 mg/L vs. 6 mg/L) has been reported in a previous study [6]. In addition, for non-intensive-care-unit hospitalized patients with severe COVID-19, C-reactive protein levels were higher (35.2 mg/L) and related to patients with moderate COVID-19 (11 mg/L).

## 4. Materials and Methods

In 2019, a 33-year-old female patient had a definitive diagnosis of AIH through a liver biopsy associated with a point score ERDHAI over fifteen based on histological and laboratory parameters defined by Brazilian Ministry of Health clinical protocol [31]. After initiating a follow-up with a hepatologist at Nossa Senhora das Graças Hospital in Paraná, Brazil, an MRI and abdominal ultrasound were done. Since the patient had reported a contact with a COVID-19-infected family member on 14 December 2020, respiratory samples (naso- and oropharyngeal) were collected from swabs and blood samples by peripheral venipuncture using hypodermic needles and sterile vacutainer tubes SST™ II Advance, BD Vacutainer (BD, Franklin Lakes, NJ, USA). The patient gave their informed consent for inclusion before participating in the study. The study was conducted in accordance with the Declaration of Helsinki, and the protocol was approved by the Ethics Committee of Oswaldo Cruz Foundation (CAAE 30468620.5.0000.5248). Samples from whole blood, serum, plasma, and swabs were sent to Laboratory of Viral Hepatitis/Fiocruz, Rio de Janeiro to perform SARS-CoV-2 RNA detection by TaqMan RT-qPCR assay (AgPath-ID™ One-Step RT-PCR, Thermo Fisher Scientific, Waltham, MA, USA) with a set of probe-associated primers (assay) aimed SARS-CoV-2 nucleocapsid (N1 and N2) and envelope (E) genes [32,33]. In addition, swab samples were submitted to viral-load quantification by RT-qPCR with SARS-CoV-2 N2 gene target from a commercial reagent (2019-nCoV_N_Positive Control; Integrated DNA Technologies, Iowa, IA, USA). SARS-CoV-2 lineage genotyping was accessed by high-throughput sequencing via MinION (Oxford Nanopore Technologies, Oxford, UK). Hepatitis B and C were investigated by serological tests based on chemiluminescence LIAISON XL murex HBsAg (DiaSorin, Sallugia, Italy) and LIAISON XL Murex HCV Ab (DiaSorin, Sallugia, Italy), respectively.

## 5. Conclusions

Given these findings, the present study represents an isolated case of AIH/COVID-19 in which a pre-existing clinical condition of CLD with fibrosis grade 2, altered laboratory parameters during SARS-CoV-2 infection, especially lymphopenia and elevated serum levels of alanine aminotransferase, and the administration of an immunosuppressor might have had an impact on the COVID-19 outcome. Here, we have highlighted the importance of physicians monitoring AIH patients’ clinical and biochemical parameters during the use of an immunosuppressor in SARS-CoV-2 infection given the possibility of poor prognostic for severe COVID-19 evolution.

## Figures and Tables

**Table 1:** Laboratory tests of an autoimmune hepatitis patient with active SARS-CoV-2 infection.

Tests	Values	Reference Values
SARS-CoV-2 PCR (swab)	Detected	
Hemoglobin (g/dL)	14.2	11.5–16
Mean corpuscular volume (μm^3^)	96.4	80–98
White blood cells (/mm^3^)	**3.120**	4.000–11.000
Segmented neutrophils (/mm^3^)	**1.342**	1.680–8.030
Band neutrophils (/mm^3^)	624	40–770
Lymphocytes (/µL)	**936**	1.000–3.990
Platelets (/mm^3^)	**142.000**	150.000–450.000
Alanine aminotransferase (IU/L)	**49**	13–39
Aspartate aminotransferase (IU/L)	36	7–52
Total bilirubin (mg/dL)	0.5	0.3–1
Indirect bilirubin (mg/dL)	0.31	0–1
C-reactive protein (mg/dL)	**12.8**	0–5
Glucose (mg/dL)	94	70–105
Urea (mg/dL)	21	17–43
Creatinine (mg/dL)	0.7	0.6–1.3

In bold, the tests that showed changes according to their respective reference values.

## Data Availability

Not applicable.
